# The Mediating Role of Metacognitive Beliefs in Self‐Compassion and Schizophrenia Recovery

**DOI:** 10.1002/brb3.71636

**Published:** 2026-08-05

**Authors:** Safia Samir Darwish, Ahlam Al‐Sarahneh, Mohammed Dibas, Aml Ali, Alaa Hamed, Abeer Barakat, Ahmed Othman, Haitham ElBoraie, Huda Hamzaa, Wafaa Ali

**Affiliations:** ^1^ Nursing Administration Department, Faculty of Nursing Menoufia University Cairo Egypt; ^2^ Faculty of Nursing Al‐Zaytoonah University of Jordan Amman Jordan; ^3^ Department of Medicine, Faculty of Medicine and Health Sciences An‐Najah National University Nablus Palestine; ^4^ Psychiatric and Mental Health Nursing Department, Faculty of Nursing Qena University Qina Egypt; ^5^ College of Nursing Northern Border University Arar Saudi Arabia; ^6^ Maternal and Newborn Health Nursing Department Faculty of Nursing Capital University Cairo Egypt; ^7^ Nursing Administration Department, Faculty of Nursing, Sohag University Sohag Egypt; ^8^ Psychiatry Department, Faculty of Medicine Helwan University Cairo Egypt; ^9^ Psychiatric and Mental Health Nursing Department, Faculty of Nursing Port Said University Port Said Egypt; ^10^ Department of Nursing, College of Applied Medical Sciences King Faisal University Al‐Ahsa Saudi Arabia

**Keywords:** mediation, metacognitive beliefs, psychiatric nursing, recovery, schizophrenia, self‐compassion

## Abstract

**Introduction:**

Schizophrenia is a chronic psychiatric disorder that significantly affects individuals’ mental health. Sustained personal recovery in schizophrenia is a multidimensional process that extends beyond symptom reduction and may be significantly influenced by psychological resources and cognitive appraisals. While self‐compassion and metacognitive beliefs are known to affect mental health outcomes independently, their interacting roles in predicting personal recovery remain unclear.

**Objective:**

This study examined the mediating role of metacognitive beliefs in the relationship between self‐compassion and recovery among individuals diagnosed with schizophrenia.

**Methods:**

A cross‐sectional correlational design was used. A purposive sample of 150 clinically stable adults diagnosed with schizophrenia (DSM‐5 criteria) was recruited from psychiatric outpatient clinics at Helwan University Hospitals, Egypt, between April and September 2024. Data were collected through structured face‐to‐face interviews using the Arabic versions of the Metacognition Questionnaire‐30, the Self‐Compassion Scale‐Short Form, and the Recovery Assessment Scale–Domains and Stages.

**Results:**

Bivariate analyses revealed a significant positive correlation between self‐compassion and personal recovery (*r* = 0.374, *p* < 0.001). While total metacognitive belief scores correlated positively with recovery (*r* = 0.346, *p* < 0.001), subscale analysis indicated this was driven primarily by the Cognitive Self‐Consciousness subscale (*r* = 0.598, *p* < 0.001). The association between self‐compassion and total metacognitive beliefs was non‐significant (*r* = 0.084, *p* = 0.304). Consequently, mediation analysis demonstrated that self‐compassion showed a significant direct association with recovery (*B* = 0.5726, *p* < 0.001); however, the indirect pathway through metacognitive beliefs was not significant (*B* = 0.0442, 95% Bootstrap CI [−0.045, 0.147]).

**Conclusion:**

Metacognitive beliefs were not found to mediate the association between self‐compassion and recovery; instead, self‐compassion was independently associated with multidimensional recovery. Clinically, these findings support the integration of self‐compassion training into long‐term schizophrenia treatment plans. Future longitudinal and intervention‐based studies are warranted to clarify the temporal dynamics of these relationships.

## Graphical abstract

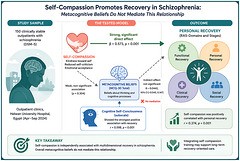

**Abbreviations**
DSM‐5: Diagnostic and Statistical Manual of Mental Disorders, Fifth Edition
MAS‐A: Metacognition Assessment Scale–Abbreviated
MCQ‐30: Metacognitions Questionnaire‐30
RAS‐DS: Recovery Assessment Scale—Domains and Stages
SCS‐SF: Self‐Compassion Scale‐Short Form
S‐REF: Self‐Regulatory Executive Function

## 1 Introduction

Schizophrenia is a severe mental disorder that affects a significant number of individuals worldwide, disrupting cognitive, emotional, and perceptual processes and impairing the ability to distinguish reality from fantasy (Meyer et al. 2011). Individuals with schizophrenia often experience difficulties in forming relationships, managing daily activities, and maintaining self‐care (Braeckman et al. 2025). Advances in treatment approaches have increasingly shifted the therapeutic focus from mere symptom management toward personal recovery (Correll et al. 2024). Recovery is now widely understood as a complex, multidimensional process that involves rebuilding identity, restoring relationships, and achieving meaningful life goals despite the persistence of the illness (Meyer et al. 2011).

A critical factor influencing this personal recovery process is metacognition. In the context of severe mental illness, metacognition is comprehensively understood through clinical frameworks such as the integrated model proposed by Lysaker et al. (2005, 2019). This model defines metacognition as a spectrum of synthetic activities that allow individuals to form complex, integrated representations of themselves and others. Deficits in this synthetic capacity are a core feature of schizophrenia spectrum disorders and represent a major barrier to functional recovery. Consequently, targeted clinical interventions have been developed to address psychological disruptions and enhance metacognitive capacity, most notably Metacognitive Reflection and Insight Therapy (MERIT) and Metacognitive Therapy (MCT) (Wiesepape et al. 2024; Lysaker et al. 2020; Wiesepape et al. 2026).

In contrast, metacognitive beliefs rooted in Wells’ Self‐Regulatory Executive Function (S‐REF) model are basically about the explicit knowledge, stances, and judgments people form regarding how they think and manage thinking. For example, this includes believing that worry is beneficial, or that some thoughts are uncontrollable, and therefore risky or even dangerous (Wells and Matthews 1996)⁠. This cognitive paradigm is examined through self‐report measures, most notably the Metacognition Questionnaire‐30 (MCQ‐30) ⁠(Wells and Cartwright‐Hatton 2004)⁠. In schizophrenia, endorsing these dysfunctional metacognitive beliefs activates the Cognitive Attentional Syndrome, characterized by persistent worry and threat monitoring, which severely interferes with psychological adjustment and recovery (Sellers et al. 2018).

In individuals with schizophrenia, self‐compassion is particularly important given the high prevalence of self‐criticism, stigma, and negative self‐perception (Mak and Cheung 2012; Feyaerts and Sass 2024). Self‐compassion promotes resilience and emotional balance by encouraging individuals to respond to personal difficulties with understanding and acceptance rather than judgment (Chan et al. 2023; Ma and Xiao 2026). From a theoretical perspective, individuals with greater self‐compassion are less likely to engage in excessive self‐criticism, worry, and maladaptive cognitive coping strategies (Eicher et al. 2013). This protective buffer may support recovery by helping individuals respond more adaptively to distressing internal experiences. Accordingly, metacognitive beliefs may represent a potential pathway linking self‐compassion to recovery in schizophrenia.

Despite advances in schizophrenia research, a significant gap remains in understanding the interplay between metacognitive beliefs and self‐compassion within the recovery process. While existing literature has explored these constructs independently, there is limited evidence regarding the mechanism through which they jointly influence recovery. Consequently, it remains unclear whether metacognitive beliefs serve as a potential pathway linking self‐compassion to clinical and personal recovery outcomes.

Nurses are uniquely positioned to address this gap, as they maintain sustained therapeutic contact with patients across outpatient and community settings (Al Barmawi et al. 2019). Through psychoeducation, therapeutic communication, and recovery‐oriented care, psychiatric nurses can develop holistic interventions that extend beyond symptom management to support long‐term recovery and psychological well‐being. Therefore, this study aimed to examine whether metacognitive beliefs may help explain the relationship between self‐compassion and recovery among individuals diagnosed with schizophrenia.

## 2 Methods

### 2.1 Setting and Study Design

The study was conducted at the psychiatric outpatient clinics of Helwan University Hospital, which provide comprehensive therapeutic services for individuals with neuropsychiatric disorders, including mental health assessments, diagnostic evaluations, medication management, and psychotherapy for selected patients. A cross‐sectional correlational design was utilized in accordance with the Strengthening the Reporting of Observational Studies in Epidemiology (STROBE) guidelines (von Elm et al. 2007).

### 2.2 Sample Size

The sample size was determined a priori using G*Power software (version 3.1.9.7) for linear multiple regression (fixed model, *F*‐test, deviation of *R* ^2^ from zero), assuming a medium effect size (*f* ^2^ = 0.15), a significance level of *α* = 0.05, a desired statistical power of 0.95, and eight predictors, including the study's primary variables and relevant sociodemographic covariates. The recruited sample of 150 participants was determined to be adequate to achieve sufficient statistical power for the regression and mediation analyses conducted.

### 2.3 Participants and Sampling

A total of 150 patients diagnosed with schizophrenia, according to the Diagnostic and Statistical Manual of Mental Disorders, Fifth Edition (DSM‐5), were recruited from Helwan University Psychiatric Hospital between April and September 2024. Participants were included if they were in a post‐acute or stable phase of illness, defined by no changes in medication, housing, or hospitalizations within the preceding 30 days. Individuals were excluded if they had comorbid psychiatric diagnoses, including mood disorders, schizoaffective disorder, or substance use disorders, or comorbid neurological and organic conditions such as intellectual disability, epilepsy, or identifiable organic brain disorders. In addition, those experiencing an acute psychotic exacerbation at the time of recruitment were also excluded. Data were collected through structured face‐to‐face interviews conducted by trained researchers to ensure consistency and reliability of the assessment process.

### 2.4 Data Collection

Data collection was carried out using structured face‐to‐face interviews conducted in private settings within psychiatric outpatient clinics to ensure confidentiality and minimize environmental distractions. The study instruments were developed based on a comprehensive review of the literature on metacognitive beliefs, self‐compassion, and schizophrenia recovery. Data collection was conducted over a 6‐month period (April–September 2024) during routine follow‐up visits.

### 2.5 Pilot Study

A pilot study involving 15 patients with schizophrenia was conducted to assess feasibility, clarity, and applicability of the tools as well as to estimate the time required for completion. No modifications were required, and pilot participants were excluded from the final sample. Each interview lasted approximately 25–30 min depending on the participant's clinical status and engagement.

### 2.6 Study Instruments and Measures

Four validated instruments were used to collect data: the Participants’ Sociodemographic and Medical Data Questionnaire, the Metacognition Questionnaire‐Short Form (MCQ‐30), the Self‐Compassion Scale‐Short Form (SCS‐SF), and the Recovery Assessment Scale–Domains and Stages (RAS‐DS). All instruments were administered in Arabic language. Translation of the original English scales followed a committee approach (Furukawa et al. 2014), involving bilingual experts, with consensus achieved on the final versions. Content validity was confirmed by a panel of experts in psychiatry, psychology, and psychiatric nursing. A pilot study with 15 participants demonstrated clarity and feasibility of the translated instruments.

The first section included a researcher‐developed questionnaire assessing participants’ sociodemographic and clinical characteristics, such as age, gender, education, marital status, income, duration of illness, and history of psychiatric hospitalization.

Metacognitive beliefs were assessed using the Arabic version of the MCQ‐30, adapted from the original scale developed by Wells and Cartwright‐Hatton (2004). The scale consists of 30 items across five dimensions: positive beliefs about worry, negative beliefs about the uncontrollability and danger of thoughts, lack of cognitive confidence, beliefs about the need to control thoughts, and cognitive self‐consciousness. Items are rated on a 4‐point Likert scale, with higher scores indicating greater endorsement of dysfunctional metacognitive beliefs. The Arabic version has demonstrated good reliability, with Cronbach's *α* coefficients ranging from 0.82 to 0.94 and an overall reliability of 0.92 (Fekih‐Romdhane et al. 2023). In the present study, the overall Cronbach's *α* was 0.75, with subscale‐level alphas ranging from 0.68 to 0.81. These values indicate acceptable reliability and reflect the expected variability in internal consistency when the instrument is applied to clinical populations with schizophrenia, characterized by heterogeneous metacognitive belief profiles, rather than to more homogeneous community samples.

Self‐compassion was measured using the 12‐item SCS‐SF, originally developed by Raes et al. (2011)⁠ from Neff's (2003)⁠ original 26‐item scale. The scale evaluates six components of self‐compassion and uses a 5‐point Likert scale. Negatively worded items are reverse‐coded, and higher scores indicate greater self‐compassion. The psychometric properties, structural stability, and reliability of the SCS‐SF have been extensively validated across diverse clinical, non‐clinical, and developmental cohorts (Bratt and Fagerström 2020; Gillett et al. 2025). In the current study, the overall Cronbach's *α* was 0.80, indicating good internal consistency.

Recovery was assessed using the 38‐item RAS‐DS, developed by Hancock et al. (2015) as an adaptation of the original Recovery Assessment Scale (RAS) by Corrigan et al. (2004). The 38‐item scale evaluates four domains: functional, personal, clinical, and social recovery, using a 4‐point Likert scale. Items are rated on a 4‐point Likert scale ranging from 1 (untrue) to 4 (completely true), with higher scores reflecting greater personal recovery. The scale has demonstrated excellent psychometric properties, with an overall Cronbach's *α* of 0.96 and strong subscale internal consistencies ranging from 0.81 to 0.94 in validation trials (Corrigan et al. 2004; Banihashemian et al. 2025). An Arabic translation of the RAS‐DS is available through the instrument's official platform. However, while no peer‐reviewed psychometric validation study of this full 38‐item version has been published to date, a recent study has successfully validated the shortened 8‐item Arabic version (RAS‐8) specifically among patients with schizophrenia (Fekih‐Romdhane et al. 2025). The overall Cronbach's *α* in the current sample was 0.95, indicating excellent internal consistency, which provides preliminary evidence of reliability for use in this Arabic‐speaking population.

### 2.7 Statistical Analysis

Statistical analyses were conducted using SPSS 26.0 (IBM, USA) to analyze the survey responses from the recruited participants. Prior to conducting regression and mediation analyses, the fundamental assumptions required for these tests were systematically examined. Multicollinearity was evaluated and ruled out, with Variance Inflation Factor (VIF) statistics falling well below acceptable thresholds. Furthermore, the assumptions of normality, homoscedasticity, and independence of residuals were assessed and deemed adequate for parametric estimation. Descriptive statistics, including frequencies, percentages, and means with standard deviations, were used to summarize participants’ general characteristics and scale scores. Pearson's correlation analysis was conducted to assess the strength and direction of associations between continuous variables. A significance threshold of *p* < 0.05 was considered statistically significant, while *p* < 0.001 was deemed highly significant. To further explore the mediating role of metacognitive beliefs in the relationship between self‐compassion and schizophrenia recovery, mediation analyses were conducted using the PROCESS macro for SPSS (Model 4) (Hayes and Rockwood 2017). A bootstrapping procedure with 5000 resamples was applied to generate bias‐corrected 95% confidence intervals for the indirect effects.

## 3 Results

**FIGURE 1:**
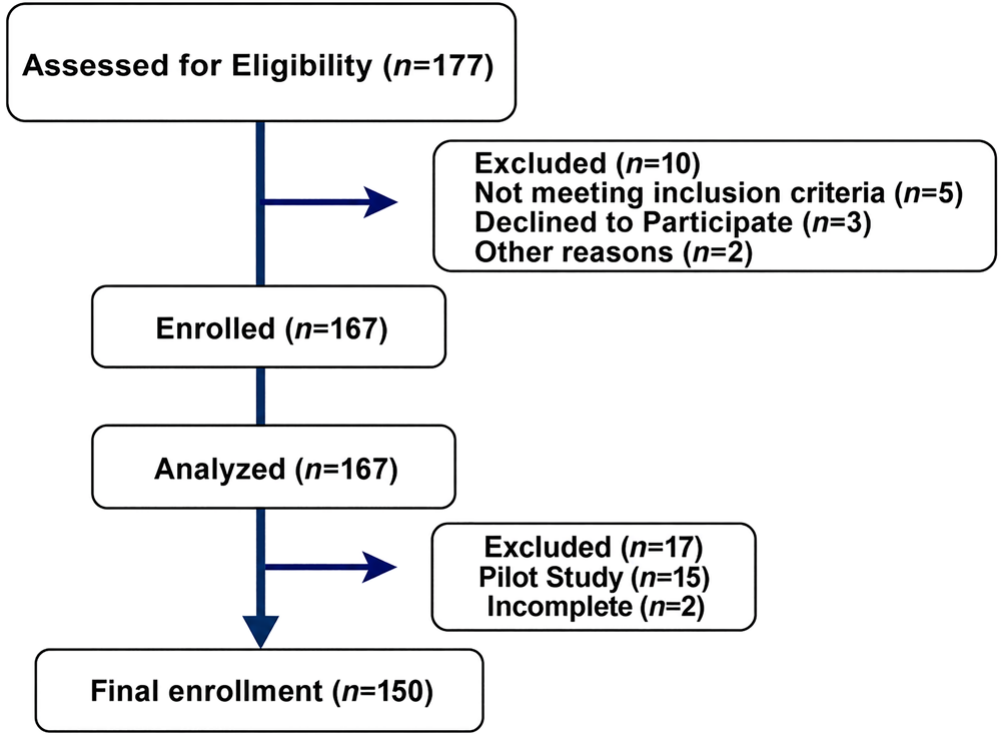
Participants’ enrollment.

Of the 177 individuals assessed for eligibility, 167 were enrolled in the study after excluding 10 individuals who did not meet the inclusion criteria, declined participation, or were excluded for other reasons. All enrolled participants were initially analyzed. Subsequently, 17 participants were excluded because they were part of the pilot study or had incomplete data, resulting in a final sample size of 150 participants. (Figure 1)

Table 1 reveals that 47.3% of the participants were under 30 years old, 69.3% were male, 44.7% were single, 57.3% lived in urban areas, 40.7% had completed primary education, and 51.3% were employed.

**TABLE 1:** Sociodemographic and clinical characteristics of participants (*n* = 150).

Factor	*n*	%
**Age**		
Less than 30 years	71	47.3
30 years–50 years	69	46.0
50 years or older	10	6.7
**Sex**		
Male	104	69.3
Female	46	30.7
**Marital status**		
Single	67	44.7
Married	61	40.7
Widowed	5	3.3
Divorced	17	11.3
**Residence**		
Rural	64	42.7
Urban	86	57.3
**Education**		
Can read and write	13	8.7
Primary education	61	40.7
Secondary education	21	14.0
University education	55	36.7
**Employment status**		
Unemployed	46	30.7
Employed	77	51.3
Student	6	4.0
Retired	21	14.0

Table 2 reveals that the mean score for metacognitive beliefs was 2.3 ± 0.3 with the following subscales: Positive Worry Beliefs (1.9 ± 0.5), Uncontrollability and Danger (2.2 ± 0.5), Cognitive Confidence (2.2 ± 0.6), Need for Control (2.2 ± 0.47), and Cognitive Self‐Consciousness (2.2 ± 0.57). Additionally, the mean score for the Self‐Compassion Scale was 3.3 ± 0.4, with its dimensions: Compassionate (2.5 ± 0.74) and Uncompassionate (4.1 ± 0.37). Furthermore, the mean score for recovery was 2.4 ± 0.6, with the following subscales: Doing Things I Value (Functional) (2.5 ± 0.87), Looking Forward (Personal) (2.4 ± 0.66), Mastering the Illness (Clinical) (2.3 ± 0.74), and Connecting and Belonging (Social) (2.41 ± 0.66).

**TABLE 2:** Scores of metacognitive beliefs, self‐compassion, and recovery among participants.

Scale	subscales	Mean ± SD	Min–Max
**Metacognitive beliefs**	Positive worry beliefs	1.9 ± 0.5	1.00–3.17
	Uncontrollability and danger	2.2 ± 0.5	1.17–3.50
	Lack of cognitive confidence	2.2 ± 0.6	1.00–3.50
	Need to control thoughts	2.2 ± 0.47	1.33–3.33
	Cognitive self‐consciousness	2.2 ± 0.57	1.17–3.83
	**Total**	**2.3** ± **0.3**	**1.47–2.93**
**Self‐compassion**	Compassionate	2.5 ± 0.74	1.33–4.17
	Uncompassionate	4.1 ± 0.37	3.17–4.83
	**Total**	**3.3** ± **0.4**	**2.75–4.08**
**Recovery**	Doing things I value (functional)	2.5 ± 0.87	1.00–4.00
	Looking forward (personal)	2.4 ± 0.66	1.22–3.78
	Mastering my illness (clinical)	2.3 ± 0.74	1.00–4.00
	Connecting and belonging (social)	2.41 ± 0.66	1.00–4.00
	**Total**	**2.4** ± **0.6**	**1.42–3.61**

Bivariate correlation analyses revealed varying patterns of association among the primary study variables. A statistically significant, moderate positive correlation was observed between self‐compassion and recovery (*r* = 0.374, *p* < 0.001) and between metacognitive beliefs and recovery (*r* = 0.346, *p* < 0.001). However, the relationship between self‐compassion and overall metacognitive beliefs was weak and did not reach statistical significance (*r* = 0.084, *p* = 0.304). At the subscale level, correlations between the five MCQ‐30 dimensions and recovery revealed differential patterns. Cognitive self‐consciousness showed the strongest positive association with recovery (*r* = 0.598, *p* < 0.001), while positive beliefs about worry (*r* = −0.031, *p* = 0.703) and lack of cognitive confidence (*r* = −0.022, *p* = 0.789) showed no significant association with recovery.

This non‐significant association between the independent variable and the mediator establishes a clear statistical rationale for the subsequent mediation analysis. The mediation analysis examined whether metacognitive beliefs explain the relationship between self‐compassion and schizophrenia recovery. As shown in Table 3, self‐compassion demonstrated a significant total effect on schizophrenia recovery (*B* = 0.617, *t* = 4.903, *p* < 0.001, 95% CI [0.368, 0.865]). When metacognitive beliefs were introduced as a mediator, the direct effect of self‐compassion on recovery remained statistically significant (*B* = 0.5726, *t* = 4.8081, *p* < 0.001, 95% CI [0.337, 0.808]). Importantly, the indirect effect of self‐compassion on recovery through metacognitive beliefs was small and statistically non‐significant (*B* = 0.0442, Bootstrap 95% CI [−0.045, 0.147]), given that the 95% bootstrap confidence interval encompassed zero. These results indicate that self‐compassion maintains a strong, direct relationship with schizophrenia recovery that is independent of metacognitive beliefs.

**TABLE 3:** Total, direct, and indirect effects of self‐compassion on recovery through metacognitive beliefs (mediation analysis).

Path model	*B*	*t*	*p*	95% CI
Total effect c (X→Y)	0.617	4.903	< 0.001	[0.368, 0.865]
Direct effect c′ (X→Y|M)	0.5726	4.8081	< 0.001	[0.337, 0.808]
Indirect effect a×b (X→M→Y)	0.0442	—	—	Bootstrap 95% CI [−0.045, 0.147]

*Note*: *X*: Self‐compassion as an independent variable; *M*: Metacognitive beliefs as mediator variable; *Y*: Recovery as a dependent variable. *B*, unstandardized regression coefficient, *t*, *t*‐statistic, *p*, probability value (*p*‐value), 95% CI, 95% Confidence Interval.

The primary mediation analysis using the total MCQ‐30 score did not support mediation (*B* = 0.044, Bootstrap 95% CI [−0.045, 0.147]). Given that the total score combines five conceptually distinct subscales with markedly different correlational patterns with recovery, an exploratory post‐hoc analysis was conducted to examine whether a specific subscale was responsible for any mediating pathway. The Cognitive Self‐Consciousness subscale (M5) was selected on the basis of its substantially stronger association with recovery (*r* = 0.598, *p* < 0.001) compared to the remaining four subscales, which showed weak or non‐significant associations. The total effect of self‐compassion on recovery was significant (c: *B* = 0.617, *p* < 0.001). After introducing M5 as the mediator, the direct effect remained significant (c′: *B* = 0.426, *p* < 0.001), and the indirect effect excluded zero (*B* = 0.191, Bootstrap 95% CI [0.058, 0.325]), supporting partial mediation, with the model accounting for 42.1% of the variance in recovery.

## 4 Discussion

The current study examined whether metacognitive beliefs function as a pathway linking self‐compassion to recovery in schizophrenia. Our findings reveal that while self‐compassion and metacognitive beliefs are both individually associated with recovery outcomes, the mediation pathway did not reach statistical significance. In other words, self‐compassion showed a significant direct association with recovery that operates independently of the metacognitive beliefs captured by the MCQ‐30.

The current study reports a significant positive association between self‐compassion and recovery among individuals with schizophrenia. This association is highly consistent with the seminal findings of Waite et al. (2015), which demonstrated that self‐compassion directly promotes psychological growth, empowerment, and a positive sense of recovery among individuals recovering from a recent psychotic episode. Similarly, the current findings corroborate the recent longitudinal and cross‐sectional evidence presented by Chan et al. (2023), which reported that higher self‐compassion is associated with reduced psychological distress, improved functioning, and enhanced well‐being. To understand the mechanism behind this direct path, it is essential to consider the unique socio‐emotional challenges that accompany a diagnosis of schizophrenia, such as self‐criticism, shame, and negative self‐evaluations.

A significant association was also observed between metacognitive beliefs and recovery. The positive correlation between total MCQ‐30 scores and recovery warrants careful interpretation, as it appears counterintuitive within the S‐REF framework where higher scores indicate greater endorsement of dysfunctional metacognitive beliefs (Wells and Matthews 1996). Subscale‐level analysis revealed that this relationship was driven primarily by the Cognitive Self‐Consciousness subscale (M5) (*r* = 0.598, *p* < 0.001), which measures the tendency to monitor and attend to one's own thinking processes. In clinically stable individuals with schizophrenia, this form of metacognitive self‐awareness may reflect preserved introspective capacity that supports engagement with recovery‐oriented goals, rather than purely pathological self‐focused attention. By contrast, the Positive Beliefs about Worry (M1) and Lack of Cognitive Confidence (M3) subscales, which more directly reflect maladaptive belief content, showed no significant association with recovery. These findings warrant further investigation, as the stronger association observed for Cognitive Self‐Consciousness suggests that the total MCQ‐30 score may partly obscure subscale‐specific differences. Future studies should examine whether Cognitive Self‐Consciousness has a distinct role in recovery using prespecified subscale‐level hypotheses and longitudinal designs.

Before interpreting the mediation findings, it is important to acknowledge a fundamental constraint of the current model. The association between self‐compassion and metacognitive beliefs was weak and non‐significant (*r* = 0.084, *p* = 0.304), meaning that the X→M pathway, a prerequisite for meaningful mediation, was not empirically established in this sample. Within Hayes’ traditional framework, the absence of a significant predictor–mediator association substantially limits the interpretive value of the mediation test, as it suggests that self‐compassion does not reliably predict metacognitive beliefs in this population (Hayes and Rockwood 2017). The non‐significant indirect effect (*B* = 0.044, 95% Bootstrap CI [−0.045, 0.147]) is therefore consistent with the earlier finding. This result could imply that, within this clinically stable schizophrenia sample, self‐compassion and MCQ‐30‐assessed metacognitive beliefs may operate as relatively separate psychological processes. Self‐compassion may support recovery more directly through reduced self‐criticism, shame, and negative self‐evaluation, rather than through changes in dysfunctional metacognitive beliefs.

A pivotal finding of the current study is that metacognitive beliefs did not significantly mediate the relationship between self‐compassion and recovery (*B* = 0.044, 95% Bootstrap CI [−0.045, 0.147]). This contrasts partially with Küçük and Tatu (2026), who reported that metacognitive beliefs directly and negatively predicted subjective recovery in schizophrenia (*β* = −0.179, *p* = 0.034), explaining 10.1% of the total variance. Notably, their model did not include self‐compassion. The current findings in our study suggest that when self‐compassion is introduced in the model, the pathway through metacognitive beliefs becomes statistically inert. This may reflect a buffering mechanism: individuals with higher self‐compassion may approach dysfunctional beliefs such as concerns about thought uncontrollability or cognitive limitations with non‐judgmental acceptance rather than self‐critical distress. Under these conditions, dysfunctional metacognitive beliefs persist but lose their clinical potency, as self‐compassion directly stabilizes the individual's relationship with distressing thoughts.

A comprehensive understanding of the current findings requires distinguishing between two conceptually distinct forms of metacognition examined in the schizophrenia literature. The MCQ‐30 measures subjective metacognitive beliefs and concerning worry about thought control (Wells and Cartwright‐Hatton 2004). In contrast, the Metacognition Assessment Scale‐Abbreviated (MAS‐A) measures objective metacognitive capacity, defined as the structural ability to form integrated representations of self and others, encompassing self‐reflectivity, understanding the minds of others, decentration, and mastery (Martiadis et al. 2023). These are fundamentally different constructs and their relationships with self‐compassion and recovery appear to operate through different mechanisms.

While subjective metacognitive beliefs (MCQ‐30) do not mediate the self‐compassion‐to‐recovery pathway, objective metacognitive capacity (MAS‐A) is structurally intertwined with self‐compassion. For instance, Hochheiser et al. (2020) demonstrated that increased metacognitive capacity, specifically the “Awareness of the Mind of the Other” subscale, uniquely and independently predicts positive self‐compassion. This implies that a certain baseline of structural metacognitive capacity must be established before an individual can successfully generate genuine self‐kindness and step back from self‐critical thoughts.

Furthermore, Bercovich et al. (2020) demonstrated that self‐compassion acts as a vital moderator in the relationship between metacognitive capacity and meaning in life. When self‐compassion is low, heightened metacognitive capacity and clinical awareness can lead to a “paradoxical effect” causing psychological pain, depression, and lower quality of life as individuals reflect on their severe difficulties. Conversely, when self‐compassion is high, individuals can safely utilize their metacognitive capacity to construct a coherent, meaningful life framework.

The current findings can therefore be situated within this broader framework. While metacognitive beliefs as measured by the MCQ‐30 did not mediate the self‐compassion–recovery pathway, consistent with the buffering mechanism discussed above, the literature suggests that structural metacognitive capacity operates differently, interacting with self‐compassion to enable safe self‐reflection and deeper recovery‐oriented functioning. Therefore, future studies should examine both constructs simultaneously to clarify their distinct and joint contributions to recovery in schizophrenia.

The exploratory post‐hoc analysis revealed that when the Cognitive Self‐Consciousness subscale (M5) was examined as a mediator in isolation, significant partial mediation was observed, with the model accounting for 42.1% of the variance in recovery. This finding suggests that the tendency to monitor and attend to one's own thinking processes, rather than the broader dysfunctional belief content captured by the total MCQ‐30 score, may represent the specific metacognitive dimension through which self‐compassion influences recovery. In clinically stable individuals with schizophrenia, cognitive self‐consciousness may reflect preserved introspective capacity that supports engagement with recovery‐oriented goals, rather than purely pathological self‐focused attention as conceptualized within the S‐REF model (Wells and Matthews 1996).

Individuals with higher self‐compassion may cultivate greater awareness of their own thinking processes, which in turn supports recovery outcomes. The direct effect of self‐compassion on recovery remained significant after introducing M5, confirming partial rather than full mediation and indicating that self‐compassion also operates through pathways beyond cognitive self‐consciousness. These findings are preliminary and require prospective replication before firm conclusions can be drawn regarding the specific role of cognitive self‐consciousness in the self‐compassion–recovery pathway.

These findings should be interpreted cautiously. Although self‐compassion emerged as a significant independent correlate of personal recovery, the snapshot nature of our data limits our ability to establish a directional pathway. Future studies utilizing longitudinal or experimental designs are warranted to further clarify the temporal dynamics of these variables and to confirm the extent to which self‐compassion serves as an antecedent to, or a concurrent facilitator of, the recovery process in this clinical population. It is also important to acknowledge that previous studies have reported variability in the strength of associations between metacognitive function and recovery outcomes, suggesting that factors such as symptom severity, illness duration, cognitive functioning, and treatment characteristics may influence these relationships. Future longitudinal studies are needed to clarify the temporal and causal pathways among self‐compassion, metacognitive beliefs, and recovery.

### 4.1 Limitations

While this study offers valuable insights into the interplay of self‐compassion, metacognitive beliefs, and recovery in individuals with schizophrenia, several limitations warrant consideration. The cross‐sectional design inherently restricts the ability to infer causality, and the focus on clinically stable outpatients may limit the generalizability of findings to more diverse or acutely ill populations. The reliance on self‐report measures introduces potential response bias, as participants may over‐ or under‐report their experiences.

Regarding the study instruments, the MCQ‐30 demonstrated reduced internal consistency in the current sample, falling below values reported in the Arabic validation study conducted with non‐clinical adults. This discrepancy suggests the MCQ‐30 may not perform optimally in clinical schizophrenia populations, potentially due to cognitive symptoms, greater heterogeneity in metacognitive belief profiles, or differential item functioning across clinical and community contexts. Additionally, no formally published psychometric validation study exists for the Arabic RAS‐DS. However, the instrument demonstrated excellent internal consistency in the current sample (*α* = 0.95).

Notably, inspection of score ranges also suggested possible restricted variability in the SCS‐SF, particularly for the total score and the reverse‐coded uncompassionate subscale. This potential ceiling effect may have attenuated associations involving self‐compassion, especially its relationship with metacognitive beliefs. Similarly, inspection of observed score ranges (Table 2) revealed a potential ceiling effect for the RAS‐DS Functional subscale, with 21.3% of participants scoring near the maximum, and a borderline floor effect for the RAS‐DS Clinical subscale (15.3% near minimum). These data characteristics may have constrained the variance available within these specific domains, potentially limiting the statistical power required to detect nuanced relationships or indirect pathways.

The mediation model did not control for several potentially important confounding variables, such as standardized symptom severity measures and antipsychotic medication regimen, representing potential uncontrolled confounders.

Despite these limitations, this study provides meaningful preliminary evidence for the direct relationship between self‐compassion and recovery in schizophrenia and highlights the independent association of metacognitive beliefs with recovery outcomes. Future longitudinal and intervention‐based studies are warranted to confirm and extend these findings, with particular attention to controlling for symptom severity and medication variables.

## 5 Conclusion

This study demonstrates that self‐compassion was independently associated with recovery among individuals diagnosed with schizophrenia, outside the indirect pathway through metacognitive beliefs. Subscale‐level analysis clarified that the counterintuitive positive correlation between metacognitive beliefs and recovery was driven primarily by cognitive self‐consciousness, which reflects preserved, adaptive introspective self‐awareness rather than pathological worry in this clinically stable outpatient sample. Integrating self‐compassion training into the long‐term treatment plan for patients with schizophrenia may support recovery by helping individuals manage distressing internal experiences, reduce self‐stigma, and alleviate harsh self‐criticism. Future longitudinal research is warranted to further clarify the temporal dynamics of these variables and confirm the directionality of the relationship between self‐reflective capacity and recovery in this clinical population.

## Author Contributions

Alaa Hamed: Writing – review and editing, validation. Safia Samir Darwish: conceptualization, methodology, software, data curation, investigation, Writing – original draft, formal analysis. Abeer Barakat: Writing – review and editing, validation. Ahmed Othman: Writing – review and editing, validation. Aml Ali: Writing – review and editing, validation. Haitham Elboraie: Writing – review and editing, validation. Mohammed Dibas: formal analysis, Writing – review and editing, data curation, project administration, methodology. Huda Hamzaa: Writing – review and editing, validation, software. Wafaa Ali: Writing – review and editing, supervision. Ahlam Al‐sarahneh: Writing – original draft, conceptualization, methodology, data curation, formal analysis, investigation.

## Funding

The authors have nothing to report.

## Ethics Statement

Ethical approval was obtained from the Ethical Research Committee, Helwan University (Session No. 40, dated 18/03/2024), in addition to official permissions from the relevant psychiatric healthcare institutions. Written informed consent was obtained from all participants prior to enrollment. All participants were informed of their right to voluntary participation and withdrawal at any time without consequences, and confidentiality was strictly maintained throughout the study.

## Conflicts of Interest

The authors declare no conflicts of interest.

## Data Availability

The corresponding author can provide the datasets used and/or analyzed for this study upon reasonable request.
